# Reduction of SARS-CoV-2 viral load in saliva after rinsing with mouthwashes containing cetylpyridinium chloride: a randomized clinical study

**DOI:** 10.7717/peerj.15080

**Published:** 2023-12-18

**Authors:** Leticia M. Bezinelli, Luciana Corrêa, Stephany Beyerstedt, Marcella L. Franco, Érika B. Rangel, Carlos Guillermo Benítez, Nelson Hamerschlak, João R.R. Pinho, Debora Heller, Fernanda P. Eduardo

**Affiliations:** 1 Hospital Israelita Albert Einstein, Sao Paulo, Brazil; 2 Universidade de São Paulo, Sao Paulo, Brazil; 3 Latin American Oral Health Association, Sao Paulo, Brazil; 4 Universidade Cruzeiro do Sul, Sao Paulo, Brazil; 5 University of Texas at San Antonio, San Antonio, TX, United States of America

**Keywords:** SARS-CoV-2, Saliva, Mouthwash, Cetylpyridinium, Zinc

## Abstract

**Background:**

Symptomatic patients with COVID-19 typically have a high SARS-CoV-2 viral load in their saliva. Procedures to reduce the viral load in their oral cavity are important for mitigating the viral transmission.

**Methods:**

This randomized clinical trial investigated the impact of two mouthwashes (0.075% cetylpyridinium chloride plus 0.28% zinc lactate (CPC+Zn) (*n* = 32), and 0.075% cetylpyridinium chloride (CPC) (*n* = 31)) on the viral load of SARS-CoV-2 in saliva when compared to the distilled water negative control (*n* = 32). Saliva was collected before (T0) and after (5 min, T1; 30 min, T2; and 60 min, T3) the intervention. Viral load in saliva was measured by qRT-PCR assays. The data in both groups was normalized for T0 and Negative Control, resulting in fold change values.

**Results:**

CPC+Zn oral solution reduced the viral load in saliva by 6.34-fold at T1, 3.6-fold at T2 and 1.9-fold at T3. Rinsing with the CPC mouthwash reduced the viral load in saliva by 2.5-fold at T1, 1.9-fold at T2 and 2.0-fold at T3.

**Conclusion:**

CPC+Zn mouthwash or with the CPC mouthwash reduced the viral load in saliva of COVID-19 patients immediately after rinsing. These reductions extended up to 60 min.

## Introduction

COVID-19 is a viral infection of high transmission, requiring controls in addition to vaccines for the mitigation of its spread. One contamination route is through saliva (Izzetti et al., 2020; Hartig et al., 2021), mainly because the SARS-CoV-2 viral load in this fluid is very high (Fernandes et al., 2020; Yoon et al., 2020; Wyllie et al., 2020; Zhu et al., 2020; Huang et al., 2021). Previous studies conducted by our group suggested that the use of mouthwash products can reduce the salivary viral load for up to 60 min (Eduardo et al., 2021; Bezinelli et al., 2023), but an effective antimicrobial solution is still under discussion. Systematic reviews analyzed the *in vitro* and *in vivo* evidence for the effects of oral antiseptics on the inactivation or eradication of SARS-CoV-2 and concluded that cetylpyridinium chloride (CPC) was the oral antiseptic with the most encouraging results (Mateos-Moreno et al., 2021; Mezarina Mendoza et al., 2022). Mouthwashes containing CPC are known to reduce oral bacteria (Hu et al., 2009; He et al., 2011) and have been used as preprocedural rinses in the dental office (Feres et al., 2010; Retamal-Valdes et al., 2017). The use of CPC against viruses has not been extensively investigated, but recent *in vitro* findings suggest that CPC mouthwashes could be effective against SARS-CoV-2 (Sarma, Gumber & Kilpatrick, 2020; Carrouel et al., 2021; Koch-Heier et al., 2021; Komine et al., 2021; Muñoz Basagoiti et al., 2021; Anderson et al., 2022; Bañó-Polo et al., 2022; Okamoto et al., 2022; Sánchez Barrueco et al., 2022; Takeda et al., 2022).

There is also some clinical evidence of the effect of CPC-containing mouthwashes on salivary SARS-CoV-2 viral load. One study showed that rinsing for 30 s using mouthwashes with 0.075% CPC reduced salivary SARS-CoV-2 levels in COVID-19 patients within 5 min, and these effects were maintained for up to 6 h (Seneviratne et al., 2021). Another study with similar conditions showed delayed reductions (after 30 and 60 min of the baseline) in the salivary SARS-CoV-2 viral load (Alzahrani et al., 2023). However, two investigations did not find a SARS-CoV-2 viral load reduction in the saliva after the use of 0.07% CPC mouthwash (Ferrer et al., 2021; Alemany et al., 2022), but a significant increase in the levels of viral particle disruption in saliva was observed after CPC oral solution exposure (Alemany et al., 2022).

Based on published *in vitro* and clinical studies, it was anticipated that rinsing with mouthwashes containing CPC would temporarily reduce the SARS-CoV-2 viral load in the saliva of hospitalized COVID-19 patients. In addition, the association of CPC with zinc could improve antiviral efficacy, a fact not explored by previous clinical studies. This could help minimize the amount of SARS-CoV-2 aerosolized from the oral cavity during routine dental and medical procedures. It could also help reduce environmental contamination by the virus in clinical offices and enhance biosafety protocols in hospitals and dental offices. Given the findings in the literature and the current pandemic/post scenario, this article aims to evaluate the reduction in viral load in saliva when mouth rinses containing cetylpyridinium chloride are used.

## Materials & Methods

### Ethical approval

The research was conducted according to the Declaration of Helsinki, revised in 2013. An informed consent form describing the study objectives and risks and benefits was signed by each patient.

### Trial design

This was a randomized, double-blind, single-center clinical trial. Two parallel intervention groups and one control group were designed to detect the equivalence of interventions in reducing the SARS-CoV-2 viral load in saliva.

### Patient eligibility

Between April 2021 and May 2021, COVID-19 inpatients at the Hospital Israelita Albert Einstein (HIAE) were considered for participation in this investigation. The study was registered at clinicaltrials.gov (NCT04902976) in May 2021. Recruitment was initiated in April 2021 after HIAE Ethical Committee approval (43550721.4.0000.0071). The study was initiated before it was registered at clinicaltrials.gov due to the difficulties in recruiting the total number of patients needed during the COVID-19 pandemic and the oscillations of hospitalizations during this period.

The inclusion criteria were age 18 to 90 years old; up to 3 days of hospitalization length and a maximum of 7 days from first symptom appearance; positivity for SARS-CoV-2 as determined by reverse transcription-polymerase chain reaction (RT–PCR) performed in nasopharyngeal samples; positivity for SARS-CoV-2 in the first collection of saliva samples; and needing oral hygiene guidance and other preventive and therapeutic oral care. The exclusion criteria were absence of SARS-CoV-2 positivity detected by RT–PCR at the time of recruitment or at the time of the first saliva collection; oral lesions that contraindicated the use of cetylpyridinium chloride mouthwash; pregnant and breastfeeding women; bleeding in the oral cavity; allergy, irritations or other side effects derived from use of the intervention solutions; exposure to intervention solutions or other antimicrobial agents 48 h before the baseline collection; disagreement with the protocol or inadequate intervention performance by the patient.

### Interventions

The following interventions were used in the study:

(a) CPC+Zn: 0.075% cetylpyridinium chloride, 0.28% zinc lactate, 225 ppm fluoride (Colgate-Palmolive Company, São Paulo, Brazil);

(b) CPC: 0.075% cetylpyridinium chloride, 225 ppm fluoride (Colgate-Palmolive Company, São Paulo, Brazil);

(c) Negative control: distilled water.

The volumes (20 mL) and rinsing times (once for 30 s) for the two test mouthwashes and for the negative control were as specified on the product label. Mouth washing was performed in the morning as the patient’s first oral hygiene procedure of the day. The participants were instructed not to perform toothbrushing or other oral hygiene procedures and were asked not to drink, eat, or chew gum after mouth washing and until the end of the experiment (for at least 60 min postrinsing). The patients were supervised by calibrated dentists to ensure compliance and to minimize experimental variations.

### Outcomes

This study measured the effects of two antimicrobial mouthwashes on their ability to reduce the viral levels of SARS-CoV-2.

### Sample size estimation

Patient sample size was determined based on previous data8. The size estimate was calculated on the standard deviation of the difference in viral load between the baseline and each experimental timepoint. A power of 90%, a two-sided significance level of 0.05, and a minimum of at least a 1-log detectable difference between each timepoint were adopted, which resulted in 30 patients in each group. Applying a 20% dropout rate, 35 patients were selected for each group.

### Randomization and blinding

Computer-generated randomization was adopted for patient allocation. After distribution into the groups, a code was assigned to each patient, allowing allocation concealment that was maintained for the patients and the laboratory researchers. The code was only revealed at the conclusion of the study after the statistical analysis was completed. Allocation in each group was performed by two senior researchers. During the study project planning phase, patient recruitment and sample collection were set to start in February 2021 and end in April 2021. However, due to a delay in the implementation of the methodological steps in the patients’ assistant routine, the recruitment and sample collections began in April 2021 and ended in May 2021.

### Saliva collection

Oral viral load was monitored based on saliva samples. For this purpose, patients were instructed by dentists, who were previously calibrated relative to saliva collection and transport. Unstimulated saliva was collected in accordance with our previous study (Eduardo et al., 2021). The time points for collection were before rinsing (T0), immediately postrinsing (within 5 min; T1), 30 min postrinsing (T2), and 60 min postrinsing (T3).

### RNA extraction and quantitative reverse transcription PCR analysis

Quantitative reverse transcription polymerase chain reaction (qRT −PCR) was used for SARS-CoV-2 viral load quantification. The nucleic acids in the saliva samples were extracted (QIASymphony DSP Virus/Pathogen kit; Qiagen, Hilden, Germany), and the RNA amount and integrity in all the samples were checked (Nanodrop™; Thermo Fisher Scientific, Waltham, MA, USA). cDNA was generated based on the manufacturer’s instructions (XGEN Master COVID-19) and as described in our previous studies (Eduardo et al., 2021; Bezinelli et al., 2023). Extracted RNA (5 µL) was added to a mixture of enzymes, probes, oligonucleotides, buffer, and deoxynucleotide triphosphates (MIX CV19, XGEN Master COVID-19). Sequences in the genes for the viral nucleocapsid (N) and polyprotein ORF1ab and human RNase P (internal control) were amplified. Negative controls for qRT −PCR were free water-only samples used for RNA elution. Positive controls were used during RNA extraction (MEGAscript T7 Transcription kit; Thermo Fisher Scientific) and during amplification (provided by a commercial kit). Viral RNA extracted from SARS-CoV-2 culture supernatant was used to generate a standard curve by diluting the samples to concentrations of 10^1^ to 10^6^ copies/mL. Thermal cycling was performed with a QuantStudio™ 6 Real-Time PCR System (Thermo Fisher Scientific) set at 45 °C for 15 min for reverse transcription, 95 °C for 2 min, 45 cycles of 95 °C for 10 s, and 60 °C for 50 s. A cycle threshold (Ct) >40 indicated that SARS-CoV-2 was undetectable. Viral load is shown in copies/mL.

### Clinical data

Data were collected as previously described (Eduardo et al., 2021). Briefly, the following patient information was collected from their medical records: sex, age, type and frequency of COVID-19-related signs and symptoms, frequency of comorbidities, extent of lung lesions, and oxygen saturation. Indices of oral health (modified gingival index and plaque index) were also registered at baseline. Additionally, the oral cavity was visually examined using a flashlight to confirm the absence of lesions in the oral mucosa.

### Statistical analyses

The double delta Ct formula ( 2^ΔΔCt^) was used for the viral fold-reduction calculation. The values for the CPC+Zn mouthwash and the CPC mouthwash at T1, T2, and T3 were normalized with the mean values of the negative control ( ΔCt Control) and with the corresponding values at T0 ( ΔCtE). A cutoff value ≥ 2 at each time point was considered a significant reduction; (Dalman et al, 2012; Zhao, Erwin & Xue, 2018). The viral load was also calculated using the standard curve and was transformed in log_10_.

Categorical and numerical clinical data were analyzed using the chi-square test and the Kruskal-Wallis test, respectively. Analysis of variance (ANOVA) was used for comparisons between the treatment groups. Within-treatment, the baseline (T0) was compared to T1, T2, and T3 using paired *t* tests. All statistical tests of hypotheses were two-sided, adopting a level of significance of = 0.05. The calculations were performed with Minitab v.18 statistical software (Minitab, LLC, State College, PA, USA).

## Results

### Participant flow and recruitment

**Figure 1:**
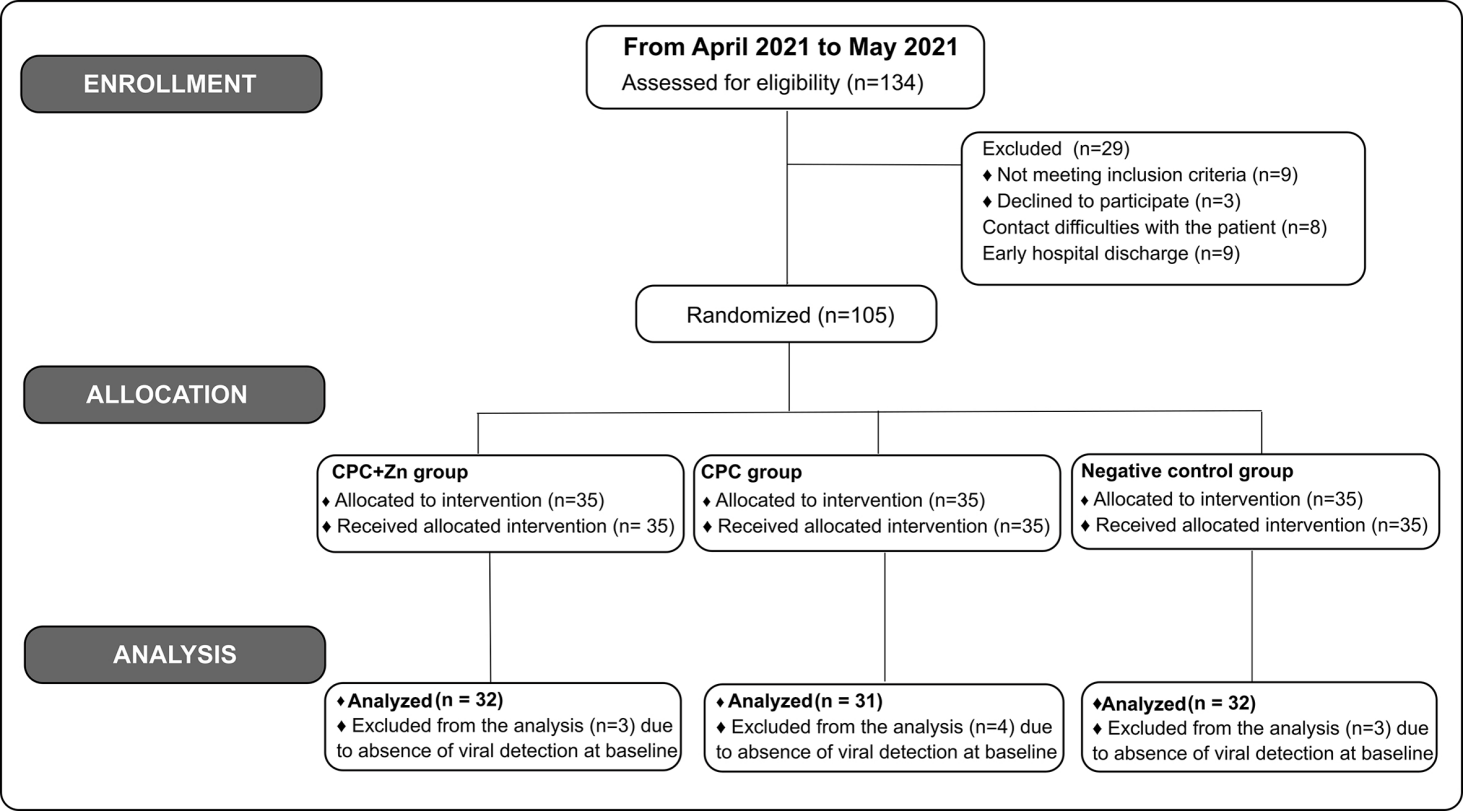
Flow diagram of the patients.

In this study, one hundred thirty-four (134) patients were screened. One hundred five (105) patients were enrolled and randomized in this study. Patients with undetectable virus levels in saliva at T0 were excluded from the final analysis study. Ninety-five (95) patients were included in the final analysis: 32 from the CPC+Zn group, 31 from the CPC group, and 32 from the negative control group (Fig. 1).

### Baseline clinical data

Recorded clinical data are shown in Table 1. Most patients were men and had a median age of 49 years (range of 23–85 years). There were no statistically significant differences among the three treatment groups with respect to age or sex. The frequencies of COVID-19-related signs and symptoms were similar across the three treatments. More than 50% of the patients in each group reported taste changes, fatigue, fever, headache, cough, nasal congestion, and dyspnea. Approximately 50% of the patients had a risk factor for COVID-19 complications, with 15–19 patients per group reporting a risk factor. The most common risk comorbidity was hypertension, followed by diabetes and cardiovascular disease. The median values of oxygen saturation varied from 93% to 94%; seven patients had oxygen saturation <90%; and five required noninvasive mechanical ventilation. A high frequency of 50% extension lung lesions was also noted, but none of the patients progressed to severe COVID-19 at the time of the study. The participants showed good oral hygiene, with only two patients with visible dental plaque; one patient showed discrete gingival inflammation. These parameters confirmed the absence of oral infection and other oral lesions in all the patients who could exert some bias in the saliva collection and analysis. There were no statistically significant differences among the groups in any of the analyzed variables.

**Table 1:** Clinical characteristics of the included patients described in the medical records.

	**CPC+Zn (*n* = 32)**	**CPC (*n* = 31)**	**Negative control (*n* = 32)**	*P*-value
Male –n (%)	22 (68.8)	18 (58.1)	19 (59.4)	0.632
Female –n (%)	10 (31.3)	13 (41.9)	13 (40.6)	
Age –median (range)	49.5 (28–85)	49 (33–65)	49 (23–85)	0.572
COVID-19 related signs/symptoms –n (%)				
Xerostomia	10 (31.3)	16 (51.6)	14 (43.8)	0.566
Smell changes	6 (18.8)	16 (51.6)	9 (28.1)	0.135
Taste changes	19 (59.4)	22 (71.0)	22 (68.8)	0.895
Fatigue	29 (90.6)	29 (93.5)	29 (90.6)	0.995
Fever (>37.5 ° C)	29 (90.6)	30 (96.8)	29 (90.6)	0.978
Headache	21 (78.1)	22 (71.0)	18 (56.3)	0.844
Coughing	25 (78.1)	23 (74.2)	27 (84.4)	0.943
Nausea	4 (12.5)	5 (16.1)	4 (12.5)	0.916
Diarrhea	1 (3.1)	5 (16.1)	3 (9.4)	0.276
Nasal congestion	23 (71.9)	22 (71.0)	21 (65.6)	0.969
Dyspnea	24 (75.0)	24 (77.4)	23 (71.9)	0.981
O_2_ saturation (%) –median (range)	93 (87–95)	94 (88–97)	94 (88–96)	0.376
Extension of lung lesion –n (%)				
<25	3 (9.4)	3 (9.7)	3 (9.4)	0.999
25	5 (15.6)	9 (29.0)	8 (25.0)	0.584
25–50	24 (75.0)	19 (61.3)	20 (62.5)	0.850
BiPAP prescription –n (%)	1 (3.1)	1 (3.2)	3 (9.4)	0.485
Patients with comorbidities –n (%)	15 (46.9)	19 (61.3)	15 (46.9)	0.764
Diabetes	5 (33.3)	3 (15.8)	4 (26.7)	
Hypertension	11 (73.3)	7 (36.8)	12 (80.0)	
Respiratory disease / asthma	1 (6.7)	1 (5.3)	0 (0.0)	
Cardiovascular disease / thrombosis	2 (13.3)	2 (10.5)	2 (13.3)	
Liver disease	2 (13.3)	0 (0.0)	0 (0.0)	
Renal disease	1 (6.7)	0 (0.0)	0 (0.0)	
Malignant neoplasms	0 (0.0)	1 (5.3)	1 (6.7)	
Obesity	1 (6.7)	0 (0.0)	1 (6.7)	
Oral health condition –median (range)				
Modified gingival index^a^	0 (0–0)	0 (0–0)	0 (0–1)	0.969
Plaque index^a^	0 (0–2)	0 (0–1)	0 (0–2)	0.563

**Notes.**

BiPAP, Bilevel positive airway pressure.

a The grades are described in the “Clinical data” section.

*P* value obtained using chi-square test and Kruskal–Wallis test.

### Fold reduction

**Figure 2:**
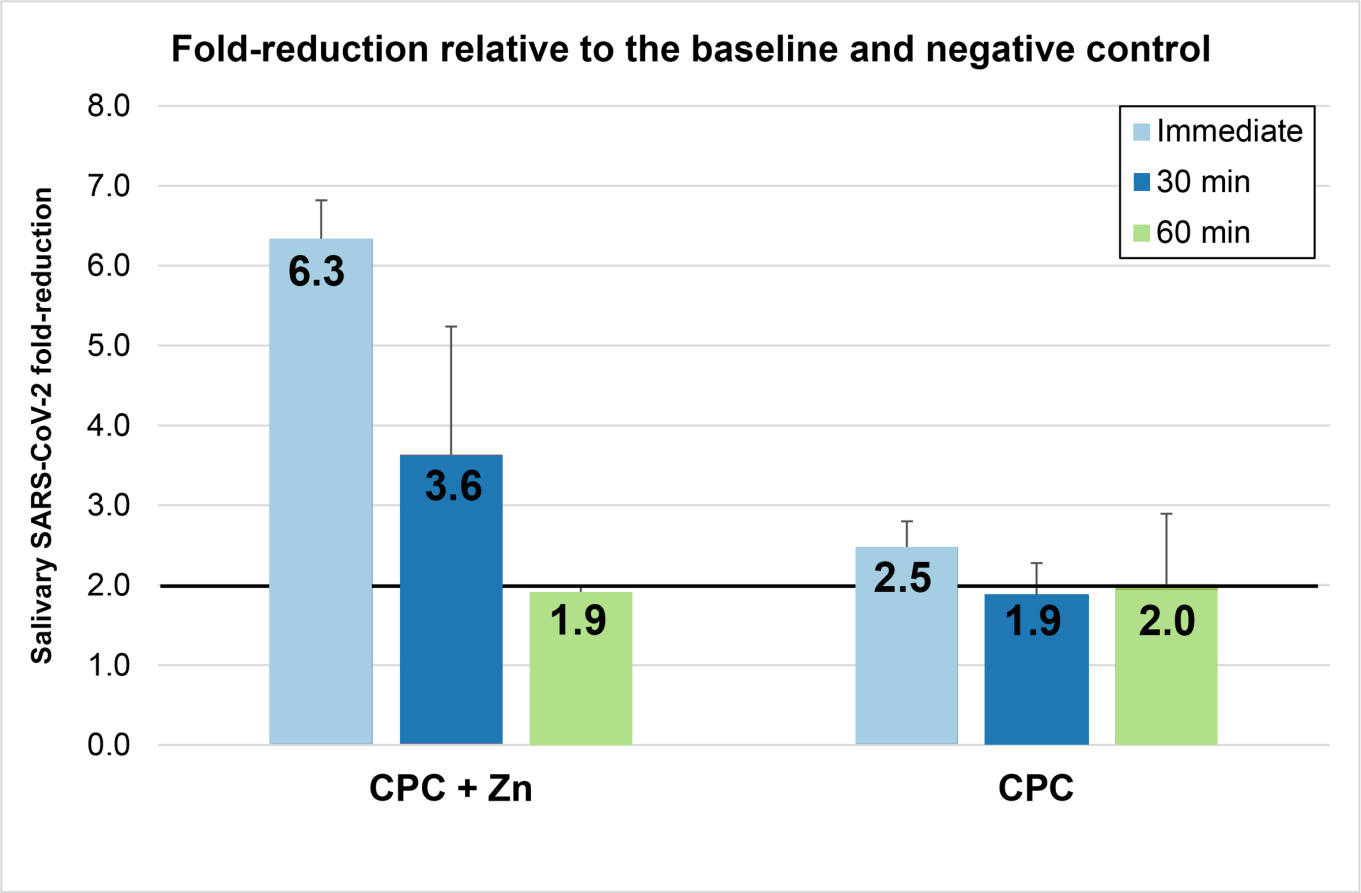
Mean (± Standard Error) of SARS-CoV-2 fold reduction in saliva immediately after rinsing (T1), 30 min after rinsing (T2), and 60 min after rinsing (T3). These fold reductions for each mouthwash are determined relative to the baseline and negative.

Fold reductions (Fig. 2) were calculated using the mean values shown in Table 2. Rinsing with the CPC+Zn mouthwash reduced the viral load in saliva by 6.34-fold at T1, 3.6-fold at T2, and 1.9-fold at T3. Rinsing with the CPC mouthwash reduced the viral load in saliva by 2.5-fold at T1, 1.9-fold at T2, and 2.0-fold at T3. At T1, the CPC+Zn and CPC groups showed fold-reductions ≥2 in saliva. The CPC+Zn intervention also met these criteria at T2 (3.64-fold change) but just missed the criteria at T3 (1.90-fold change). Conversely, the CPC intervention just missed the criteria at T2 (1.89-fold change) but did meet the criteria at T3 (2.01-fold change).

**Table 2:** Cycle threshold (Ct) values in the saliva of COVID-19-positive patients treated with mouthwashes at baseline (T0), immediately after rinsing (T1), 30 min after rinsing (T2), and 60 min after rinsing (T3).

**Treatment**	*n*	**Baseline** **Mean ± SD**	**Immediate** **Mean ± SD**	**30-minute** **Mean ± SD**	**60-minute** **Mean ± SD**
CPC+Zn	32	29.03 ± 4.72	31.67 ± 5.46	30.27 ± 5.25	29.29 ± 3.80
CPC	31	29.61 ± 5.57	30.89 ± 6.07	29.90 ± 4.93	29.95 ± 5.28
Negative control	32	30.31 ± 4.60	30.28 ± 4.11	29.68 ± 4.72	29.64 ± 6.41

### Viral load

The mean SARS-CoV-2 viral loads (in log10) are shown in Tables 3 and 4. In the CPC+Zn and CPC groups, the viral loads were reduced at T1, T2, and T3 compared to baseline (Table 3). In the negative control, there was a trend toward an increase in the mean viral load from baseline to T3. The viral load reductions were statistically significant at T1 in the two test groups (Table 4).

**Table 3:** SARS-CoV-2 viral load (log_10_) at baseline (T0), immediately postrinsing (T1), 30 min postrinsing (T2) and 60 min postrinsing (T3) for patients who completed the clinical study.

**Treatment** **(subjects)**	**Baseline** **Mean ± SD** **(%Change *vs* baseline)**	**Immediate** **Mean ± SD** **(%Change *vs* baseline)**	**30-minute** **Mean ± SD** **(%Change *vs* baseline)**	**60-minute** **Mean ± SD** **(%Change *vs* baseline)**
CPC+Zn (*n* = 32)	3.01 ± 1.45 (–)	2.29 ± 1.38 (−80.9%)	2.72 ± 1.29 (−48.7%)	2.92 ± 1.18 (−18.7%)
CPC (*n* = 31)	2.96 ± 1.66 (–)	2.67 ± 1.55 (−48.7%)	2.86 ± 1.48 (−20.6%)	2.87 ± 1.53 (−18.7%)
Negative control (*n* = 32)	2.63 ± 1.48 (–)	2.63 ± 1.35 (0.0%)	2.80 ± 1.52 (33.9%)	2.92 ± 1.73 (48.7%)

**Table 4:** Baseline-adjusted SARS-CoV-2 viral load (log_10_) at the immediate postrinsing (T1), 30-minute postrinsing (T2) and 60-minute postrinsing (T3) examinations for patients who completed the clinical study.

Treatment	n	Adj Immediate Mean ± SE	Adj. 30-Minute Mean ± SE	Adj. 60-Minute Mean ± SE
CPC+Zn	32	2.17 ± 0.123^a^	2.60 ± 0.133	2.81 ± 0.170
CPC	31	2.60 ± 0.125^b^	2.78 ± 0.135	2.80 ± 0.172
Negative control	32	2.82 ± 0.123	2.99 ± 0.133	3.09 ± 0.170

**Notes.**

a Difference between baseline and immediate post-rinsing examinations is statistically significant at *p* < 0.001.

b Difference between baseline and immediate post-rinsing examinations is statistically significant at *p* = 0.016.

### Adverse events

Adverse events were not observed after the interventions.

## Discussion

In this study, we showed that CPC and CPC+Zn mouthwashes reduced the SARS-CoV-2 viral load in the saliva of COVID-19 inpatients. The antiviral potential of CPC reported by other studies (Seneviratne et al., 2021; Alzahrani et al., 2023) was confirmed in the current clinical study. While the mechanism by which these solutions reduce the viral load in the oral cavity still needs to be elucidated, it is postulated that the viral load within the mouth is reduced through physicochemical adulteration of the viral envelope. According to some *in vitro* studies, CPC demonstrated significant virucidal activity against SARS-CoV-2 through disruption of the viral envelope (Muñoz Basagoiti et al., 2021). Integration of oral antiseptics into the viral envelope will result in its permeabilization, ultimately resulting in virus neutralization.

In a previous pilot study, our group showed that CPC mouthwash reduced the SARS-CoV-2 viral load in saliva for up to 60 min, a time interval considered sufficient for reducing the contamination risk during dental procedures in SARS-CoV-2-positive patients (Eduardo et al., 2021). This current clinical study was performed with a larger sample and confirmed this previous finding. These results are in agreement with those reported by other authors (Seneviratne et al., 2021), who also postulated that these formulations may be used as preprocedural rinses to help reduce the transmission of COVID-19. In addition, the current study demonstrated, for the first time, that the association of CPC with zinc also temporarily reduced the SARS-CoV-2 salivary viral load among COVID-19 patients.

The role of zinc in controlling SARS-CoV-2 requires further investigation. In vitro and clinical studies suggest that this ion has antiviral properties against herpes virus, respiratory syncytial virus, rhinovirus (Read et al., 2019), SARS-CoV (te Velthuis et al., 2010) and SARS-CoV-2 (Saadh et al., 2021), among other viruses, but the mechanisms of action of these antiviral properties need to be elucidated. An additional review concluded that zinc ion availability is important for efficacy measurement (Eby, 1997).

An unexpected finding in the current study was a trend of viral load increase in the negative control (mouth washing with only distilled water) 60 min postrinsing, although this difference was not statistically significant in relation to the baseline. We interpreted this result as natural virus kinetics in the saliva in accordance with the circadian cycle, considering that in all the patients, the mouth washing and saliva collection were performed in the morning, immediately after the patient’s awakening. A study showed that from 4 a.m. to 12 p.m., there is high viral shedding in the saliva in comparison with the evening period (Viloria Winnett et al., 2022); this fact is associated with natural salivary flow stimulation after awakening, which may explain the slight increase in viral load after mouth washing with water. Other studies focused on the effects of oral antimicrobial solutions on the salivary SARS-CoV-2 viral load (Ferrer et al., 2021; Sánchez Barrueco et al., 2022; Alzahrani et al., 2023) showed a decrease in viral load after mouth washing with water, attributing this finding to the mechanical action of rinsing (Sánchez Barrueco et al., 2022; Alzahrani et al., 2023). Some differences in the study design between our study and those of the referenced authors, especially in relation to the time of the experimental procedure (only one of these studies mentioned that the experimental procedure was performed in the morning), may explain this discrepancy.

It is important to mention that the current study cannot differentiate the impact of the mouthwashes relative to the viral origin source, although presumably the bulk of viruses neutralized are found in saliva. The impact of the oral solutions on SARS-CoV-2 infectivity must also be further analyzed, although the significant viral load reductions observed mainly at T1 and in the *in vitro* studies have demonstrated an important CPC antiviral potential. Another important limitation was that this study included only inpatients with signs and symptoms of COVID-19. The interferences of these variables in the saliva were not controlled, which limits the extrapolation of these results to asymptomatic patients.

Another question not addressed in the current study but with important clinical implications is the CPC mouthwash effect (with or without zinc) on the oral microbiome as a whole. Daily use of CPC mouthwash can induce changes in the oral microbiome, such as reductions in microbial diversity and gingivitis-related bacterial abundance, which can lead to positive clinical outcomes in patients with gingivitis and periodontitis (do Amaral et al., 2023). Moreover, some studies have shown that in COVID-19-positive patients, salivary bacterial diversity is reduced in relation to that in negative patients (Iebba et al., 2021; Ren et al., 2021), although larger investigations are necessary to confirm these findings (Miller et al., 2021). Therefore, comprehensive studies analyzing the CPC effect on the SARS-CoV-2–bacterial relationship must be conducted to improve mouthwash protocols and avoid unexpected side effects.

Despite the temporary effects of mouthwashes, the current study reinforces the findings of our pilot study and provides encouraging results regarding the use of these products as preprocedural rinses for patients infected with SARS-CoV-2 and may be used as a risk mitigation practice for asymptomatic patients. The use of mouthwashes as an adjunct to current mitigation practices, such as the use of masks, handwashing, disinfection, and social distancing efforts, should be considered. In addition, the incorporation of a preprocedural rinse prior to any dental procedures would help create a safer working environment for dental professionals by reducing their exposure to pathogens such as SARS-CoV-2 during routine dental procedures.

## Conclusions

The current study demonstrated that the CPC and CPC+Zn oral solutions reduced the viral load in the saliva of COVID-19 patients immediately after rinsing. These reductions extended up to 60 min.

##  Supplemental Information

10.7717/peerj.15080/supp-1 Supplemental Information 1 Raw data

10.7717/peerj.15080/supp-2 Supplemental Information 2 Raw Clinical Data

10.7717/peerj.15080/supp-3 Supplemental Information 3 Clinical protocol

## Additional Information and Declarations

### Data Availability

The following information was supplied regarding data availability: The raw data are available as Supplementary Files.

### Clinical Trial Registration

The following information was supplied regarding Clinical Trial registration: NCT04537962
