# Supplementary material for: Reduction of SARS-CoV-2 viral load in saliva after rinsing with mouthwashes containing cetylpyridinium chloride: a randomized clinical study
Source: PeerJ. 2023 Dec 18;11:e15080. doi: 10.7717/peerj.15080 (PMC10734404; doi:10.7717/peerj.15080)
Supplement: Supplemental Information 3 [file peerj-11-15080-s003.pdf]

ClinicalTrials.gov PRS
Protocol Registration and Results System

ClinicalTrials.gov PRS DRAFT Receipt (Working Version)
Last Update: 02/23/2022 11:34

ClinicalTrials.gov ID: NCT04537962

Study Identification

Unique Protocol ID: 4172-20

Brief Title: Salivary SARS-CoV-2 Load of Covid-19 Patients After Oral Antimicrobial Solutions and Dentifrices

Official Title: Avaliação da Carga do vírus SARS-CoV-2 na Cavidade Oral e na Saliva após desinfecção Com soluções Antimicrobianas Orais e dentifrícios.

Secondary IDs:

Study Status

Record Verification: July 2020

Overall Status: Completed

Study Start: July 14, 2020 [Actual]

Primary Completion: December 30, 2020 [Actual]

Study Completion: September 30, 2021 [Actual]

Sponsor/Collaborators

Sponsor: Hospital Israelita Albert Einstein

Responsible Party: Sponsor

Collaborators: Colgate Palmolive

Oversight

U.S. FDA-regulated Drug: No

U.S. FDA-regulated Device: No

U.S. FDA IND/IDE: No

Human Subjects Review: Board Status: Approved
Approval Number: 4270-20
Board Name: Titulação de IgA e IgG salivar anti-SARS-CoV-2 segundo o tempo e a gravidade de COVID-19: estudo piloto
Board Affiliation: HIAE
Phone: 55 11 2151-1386
Email: cep@einstein.br
Address:

Av. Albert Einstein 627
São Paulo

Brasil
05652-900

Data Monitoring: Yes

FDA Regulated Intervention: No

Study Description

Brief Summary: The aim of this study is to analyze if the use of oral antimicrobial solutions and dentifricies are able to reduce the SARS-CoV-2 load in the saliva and oral mucosa. It will be allocated hospitalized patients positive for SARS-CoV-2 (confirmed by RT-PCR of nasopharynx swab tests) and with signs and symptoms of COVID-19. These patients will be divided into two groups: patients enrolled in negative pressure rooms (NPR), and patients enrolled in intensive care units (ICU) with orotracheal intubation. These two groups will receive interventions with oral antimicrobial solutions or dentifrices, containing different compounds. Saliva and oral mucosa swabs will be collected before the intervention, immediately after the intervention, and after 30min and 1h. The primary outcome is to verify if these products can reduce the SARS-CoV-2 load in the saliva and oral mucosa at these time periods, detected by the measurement of the viral load and the fold-reduction.

Detailed Description: Each group of patients will receive specific interventions, as follows:

NPR group (n=60) – three interventions with different mouthwashes solutions (12 patients in each intervention): 1) 0.12% chlorhexidine solution (Colgate Periogard®); 2) mouthwash with 1.5% hydrogen peroxide solution (Colgate Peroxyl®); 3) mouthwash with 0.075% cetylpyridinium chloride solution (Colgate Total 12®); 4) 1.5% hydrogen peroxide solution plus 0.12% chlorhexidine solution (Colgate Peroxyl® followed by Colgate Periogard®).

The interventions will be compared with a Placebo, which will be a mouthwash with distilled water (n=12).

NPR group (n=90) – three interventions with different dentifrices (30 patients in each intervention): 1) dentifrice containing only 1.1% fluoride, water, glycerin, cellulose, sodium lauryl sulfate, and sodium bicarbonate; 2) dentifrice containing 0.32% fluoride, 0.96% zinc, arginine, poloxamer, glycerin, water, hydrated silica, sodium lauryl sulfate, and sodium saccharin 3) dentifrice containing 0.454% stannous fluoride, water, sorbitol, hydrated silica, glycerin, tetrasodium pyrophosphate, microcrystalline cellulose, and xanthan gum .

The interventions will be compared to each other. ICU group (n=52)– two interventions with different oral antimicrobial solutions (26 patients in each intervention): 1) 0.12% chlorhexidine solution (Colgate Periogard®); 2) 1.5% hydrogen peroxide solution plus 0.12% chlorhexidine solution (Colgate Peroxyl® followed by Colgate Periogard®).

The interventions will be compared to each other.

Conditions

Conditions: Corona Virus Infection

Keywords:

Study Design

Study Type: Interventional

Primary Purpose: Supportive Care

Study Phase: N/A

Interventional Study Model: Factorial Assignment

Number of Arms: 7

Masking: Triple (Participant, Care Provider, Outcomes Assessor)

Allocation: Randomized

Enrollment: 202 [Actual]

Arms and Interventions

Arms	Assigned Interventions
Placebo Comparator: Colgate Periogard and Peroxyl®<br>Patients hospitalized in the ICU with orotracheal intubation or negative pressure room - will undergo antisepsia of the oral mucosa with 1.5% hydrogen peroxide solution, following by a 0.12% non-alcoholic chlorhexidine solution	Colgate Periogard® mouthwash<br>Patients will be submitted to antisepsia of the oral mucosa with Colgate Periogard® mouthwash<br>Colgate Peroxyl® mouthwash<br>Patients will be submitted to antisepsia of the oral mucosa with Colgate Peroxyl® mouthwash
Placebo Comparator: Colgate Periogard®<br>Patients hospitalized in the ICU with orotracheal intubation or negative pressure room - will undergo antisepsia of the oral mucosa with 0.12% non-alcoholic chlorhexidine solution;	Colgate Periogard® mouthwash<br>Patients will be submitted to antisepsia of the oral mucosa with Colgate Periogard® mouthwash
Placebo Comparator: Colgate Peroxyl®<br>Patients hospitalized in negative pressure rooms - will undergo antisepsia of the oral mucosa with 1.5% hydrogen peroxide solution	Colgate Peroxyl® mouthwash<br>Patients will be submitted to antisepsia of the oral mucosa with Colgate Peroxyl® mouthwash
Placebo Comparator: Colgate Total 12®<br>Patients hospitalized in negative pressure rooms - will undergo antisepsia of the oral mucosa with 0.075% cetylpyridinium chloride associated with 0.28% zinc lactate	Colgate Total® Mouthwash<br>Patients will be submitted to antisepsia of the oral mucosa with Colgate Total® Mouthwash
Active Comparator: Toothpaste with sodium monofluorophosphate<br>Patients hospitalized in negative pressure rooms - will undergo brushing with dentifrice containing only 1.1% fluoride, water, glycerin, cellulose, sodium lauryl sulfate, and sodium bicarbonate	Toothpaste with sodium monofluorophosphate<br>Patients will be submitted to brushing with toothpaste with sodium monofluorophosphate
Active Comparator: Toothpaste with sodium fluoride and zinc<br>Patients hospitalized in negative pressure rooms - will undergo brushing with dentifrice containing 0.32% fluoride, 0.96% zinc, arginine, poloxamer, glycerin, water, hydrated silica, sodium lauryl sulfate, and sodium saccharin	Toothpaste with sodium fluoride and zinc<br>Patients will be submitted to brushing with toothpaste with sodium fluoride and zinc
Active Comparator: Toothpaste with tin fluoride<br>Patients hospitalized in negative pressure rooms - will undergo brushing with dentifrice containing 0.454% stannous fluoride, water, sorbitol, hydrated silica, glycerin, tetrasodium pyrophosphate, microcrystalline cellulose, and xanthan gum	Toothpaste with tin fluoride<br>Patients will be submitted to brushing with toothpaste with tin fluoride

Outcome Measures

Primary Outcome Measure:

1. Reduction of SARS-CoV-2 load in the oral mucosa and saliva
   Reduction of SARS-CoV-2 load in the oral mucosa and saliva measured by viral fold-reduction and viral quantitation

   [Time Frame: 1 hour]

Eligibility

Minimum Age: 18 Years

Maximum Age: 90 Years

Sex: All

Gender Based: No

Accepts Healthy Volunteers: No

Criteria: Inclusion Criteria:

- Patients positive for SARS-CoV-2 using the RT-PCR method and requiring oral hygiene care and other preventive and therapeutic dental procedures.

Exclusion Criteria:

- Pediatric patients, negative for SARS-CoV-2 by the RT-PCR method, exhibiting oral ulcerations and other erosive lesions in the oral mucosa that contraindicate the use of hydrogen peroxide, chlorhexidine and cetylpyridinium, patients who present bleeding in the oral cavity. that prevents the collection of samples, patients who report a history of allergy, irritations or other side effects derived from the use of these substances, who do not adhere to the oral care protocols or those in which it is not possible to perform these procedures.

Contacts/Locations

Central Contact Person: Fernanda P Eduardo, PhD
Telephone: +5511999037553 Ext. 55
Email: fpeduard@einstein.br

Central Contact Backup: Leticia M Bezinelli, PhD
Telephone: 5511991589350
Email: lebezinelli@einstein.br

Study Officials: Luciana Correa, PhD
Study Director
University of Sao Paulo

Debora H Douek, PhD
Study Chair
Hospital Israelita Albert Einstein

Locations: **Brazil**
Hospital israelita Albert Einstein
Sao Paulo, Brazil, 05652-900
Contact: Fernanda P Eduardo, Phd 11999037553
Contact: Leticia M Bezinelli, PhD 5511991589350

IPDSharing

Plan to Share IPD:

References

Citations:

Links:

Available IPD/Information:
